# The interleukin-20 receptor axis in early rheumatoid arthritis: novel links between disease-associated autoantibodies and radiographic progression

**DOI:** 10.1186/s13075-016-0964-7

**Published:** 2016-03-11

**Authors:** Tue Wenzel Kragstrup, Stinne Ravn Greisen, Morten Aagaard Nielsen, Christopher Rhodes, Kristian Stengaard-Pedersen, Merete Lund Hetland, Kim Hørslev-Petersen, Peter Junker, Mikkel Østergaard, Malene Hvid, Thomas Vorup-Jensen, William H. Robinson, Jeremy Sokolove, Bent Deleuran

**Affiliations:** Department of Biomedicine, Aarhus University, Aarhus, Denmark; Department of Immunology and Rheumatology, Stanford University, Stanford, CA USA; Department of Rheumatology, Aarhus University Hospital, Aarhus, Denmark; Copenhagen Center for Arthritis Research, Center for Rheumatology and Spine Diseases, Rigshospitalet and Glostrup Hospital, Glostrup, Denmark; Department of Clinical Medicine, Faculty of Health and Medical Sciences, University of Copenhagen, Copenhagen, Denmark; University of Southern Denmark, Odense, Denmark; Odense University Hospital, Odense, Denmark; Department of Clinical Medicine, Aarhus University, Aarhus, Denmark

**Keywords:** Cytokines, Rheumatoid arthritis, Autoantibody(ies), Bone resorption, Monocytes/macrophages

## Abstract

**Background:**

Rheumatoid arthritis (RA) is often characterized by the presence of rheumatoid factor, anti-citrullinated protein antibodies, and bone erosions. Current therapies can compromise immunity, leading to risk of infection. The interleukin-20 receptor (IL-20R) axis comprising IL-19, IL-20, and IL-24 and their shared receptors activates tissue homeostasis processes but not the immune system. Consequently, modulation of the IL-20R axis may not lead to immunosuppression, making it an interesting drug target. We evaluated the role of the IL-20R axis in RA and associations between plasma cytokine levels and clinical disease.

**Methods:**

Plasma IL-19, IL-20, and IL-24 levels were measured in early RA patients during a treat-to-target strategy by enzyme-linked immunosorbent assays. The IL-20R1 and IL-22R1 levels in paired peripheral blood mononuclear cells and synovial fluid mononuclear cells from a different cohort of RA patients were evaluated by flow cytometry and confocal microscopy. Monocytes/macrophages were stimulated with heat-aggregated human immunoglobulin immune complexes and immune complexes containing citrullinated fibrinogen, and osteoclasts were incubated with IL-19, IL-20, and IL-24.

**Results:**

The plasma concentrations of IL-20 and IL-24 (but not IL-19) were increased in early RA patients compared with healthy controls (both *P* < 0.002) and decreased after 6 months of treatment (both *P* < 0.0001). The expression of IL-22R1 (but not IL-20R1) was increased on monocytes from RA synovial fluid compared with monocytes from both RA and healthy control peripheral blood. The plasma concentrations of IL-20 and IL-24 were increased in rheumatoid factor and anti-citrullinated protein antibody positive compared with negative early RA patients (all *P* < 0.0001). Immune complexes stimulated the production of the IL-20R cytokines by monocytes/macrophages. Increased baseline plasma concentrations of IL-20 and IL-24 were associated with Sharp-van der Heijde score progression after 24 months (Spearman’s rho = 0.19 and 0.26, both *P* < 0.05) in the early RA patients. The IL-22R1 was expressed by osteoclast precursors and in multinucleated osteoclasts. IL-20 and IL-24 increased the secretion of monocyte chemoattractant protein 1 by these cells.

**Conclusions:**

This study suggests that IL-20 and IL-24 link RA-associated autoantibodies with radiographic progression via the IL-22R1. Modulation of this axis holds promise as feasible anti-erosive treatment modalities in seropositive RA.

## Background

Rheumatoid arthritis (RA) is a chronic immune-mediated inflammatory disease. Current therapies are aimed at halting erosive joint progression through sustained synovitis suppression. However, these treatments can compromise the normal immune response. This results in an increased risk of infection, which is the main concern of biological agents such as anti-tumor necrosis factor alpha (anti-TNFα) [1].

The interleukin (IL)-20 receptor (IL-20R) axis is pivotal for epithelial tissue homeostasis, but is generally not assumed to directly activate cells of the immune system as a main function [2–4]. Consequently, modulation of the IL-20R axis may not lead to immunosuppression, making it an interesting treatment option [5, 6]. The IL-20R axis consists of the three cytokines IL-19, IL-20, and IL-24 (“the IL-20R cytokines”) and their two shared receptor complexes. All three bind the receptor complex of IL-20R2/IL-20R1, while IL-20 and IL-24 also bind the receptor complex of IL-20R2/IL-22R1 [7, 8]. Recently, the IL-20R2 subunit was identified as a novel risk locus for the development of RA [9], suggesting the IL-20R axis could be implicated in the pathogenesis of RA.

RA is often characterized by high levels of rheumatoid factor (RF) and anti-citrullinated protein antibodies (ACPAs) in serum [10]. RF and ACPAs are autoantibodies targeting the Fc portion of immunoglobulin (Ig)G and citrullinated proteins, respectively [1]. Both of these autoantibodies contribute to the formation of immune complexes (ICs); thus, they potentially contribute to the disease process in RA, e.g. by stimulating monocytes/macrophages through Fc receptors (FcRs) [11]. Furthermore, ICs containing citrullinated fibrinogen (cFb) were recently shown to co-stimulate macrophages via dual engagement of Toll-like receptor 4 (TLR4) and the Fcγ receptor IIa (FcγRIIa) [12].

The hallmark of RA joint disease is the development of bone erosions, which are mediated by osteoclasts (OCs) [1]. The OCs are tartrate-resistant acid phosphatase positive (TRAP^+^) multinucleated cells capable of resorbing bone [13]. The OC precursors can be found among receptor activator of nuclear factor kappa-B positive (RANK^+^) monocytes and also express the receptor for the chemokine monocyte chemoattractant protein 1 (MCP-1) (also known as C–C chemokine ligand 2) [14–16]. In RA, OC formation and activity are closely associated with the local cytokine milieu. In line with this, OCs can be generated spontaneously in vitro from RA synovial cells [17].

The IL-20R axis has been associated with several immune-mediated inflammatory diseases including psoriasis, inflammatory bowel disease, and arthritis [4, 18–25]. In the context of arthritis, it is intriguing that IL-20 has also been reported as a driver of osteoporosis [26] and that both IL-19 and IL-24 had roles in a rat model of bone resorption [27]. Recently, neutralization of IL-20 in a phase IIa trial was effective in treating seropositive RA patients, suggesting a link between IL-20 and RF [28]. In expression profile studies, production of the IL-20R cytokines was found in TLR4-stimulated monocytes/macrophages, while the receptor subunits were primarily identified on epithelial cells of target organs (e.g., skin, gut, and bone) [2, 3, 29]. However, the cellular sources and targets of the IL-20R cytokines in RA are not well understood.

Previously, we found IL-20 and IL-24 protein in the synovial membrane and synovial fluid of patients with chronic RA, and that plasma IL-20 and IL-24 levels correlated with MCP-1 [20]. The aim of this study was to determine the role of the IL-20R axis in early RA, with a focus on the associations of IL-19, IL-20, and IL-24 with clinical disease parameters and prognosis.

## Methods

### Study subjects

Plasma samples from patients with early treatment naïve RA were obtained from the OPERA study. A random set of 152 samples at baseline and after 6 months of treatment was used for measuring the plasma concentration of IL-19, IL-20, and IL-24 (Table 1). A detailed study design has been published elsewhere [30, 31]. At the entry of the double-blinded study, patients were randomized to conventional therapy with methotrexate and intra-articular steroid injections or conventional therapy combined with adalimumab. Adalimumab was discontinued after 1 year of treatment, but reinitiated when there was disease relapse. Clinical disease parameters and test results, including radiographs, were registered at the initiation of treatment and after 6, 12, and 24 months. Radiographic progression was scored according to the Sharp-van der Heijde method [32]. Radiographic outcomes were the progression from baseline and the smallest detectable change was 1.83 total Sharp score (TSS) units. The percentage of progressors were the percentage of patients with change in TSS ≥1.83 TSS units. No cells were collected from this patient cohort. In another study population consisting of chronic RA patients with at least one swollen joint (for obtaining synovial fluid), paired synovial fluid mononuclear cells (SFMCs) and peripheral blood mononuclear cells (PBMCs) were analyzed by flow cytometry and OCs were cultured from SFMCs (n = 15). Only diagnosis was recorded for this study population, as these cells were only used in vitro for proof of principle experiments.

**Table 1:** Patient characteristics

	Early RA				HC
	(n = 152)				(n = 88)
Time after treatment initiation (months)	0	6	12	24	
Age (years)	56 (43–63)	–	–	–	53 (46–61)
Gender (% female)	69	–	–	–	68
Baseline characteristics					
Disease duration (days)	84 (43–130)	–	–	–	–
IgM-RF (% positive)	71	–	–	–	–
Anti-CCP antibody (% positive)	65	–	–	–	–
Treated with adalimumab (%)	50	–	–	–	–
Disease activity					
HAQ (0–3)	1.1 (0.8–1.8)	0.1 (0–0.6)	0.1 (0–0.5)	0.1 (0–0.5)	–
DAS28CRP (0–10)	5.6 (4.9–6.3)	2.4 (1.8–3.0)	2.0 (1.8–2.8)	2.0 (1.8–2.7)	–
Radiographic progression					
Total Sharp score (% progressors)	–	32	41	48	–

Data are expressed as the median with interquartile range unless otherwise indicated. *CCP* Cyclic citrullinated peptide, *DAS28CRP* disease activity score 28 based on C-reactive protein, *HC* healthy control, *IgM* immunoglobulin M, *RF* rheumatoid factor, *HAQ* health assessment questionnaire, *RA* rheumatoid arthritis

Plasma samples from age- and gender-matched healthy controls (HCs) from the Donor Bank at Aarhus University Hospital (n = 88) were included for measuring the plasma concentration of IL-19, IL-20, and IL-24 (Table 1). The PBMCs or monocyte-derived macrophages from HCs were included for flow cytometric analysis and IC stimulation assays. Buffy coats were collected from the Donor Bank at Aarhus University Hospital (n = 10) or Stanford Blood Center (n = 2).

### Ethics

All clinical samples were obtained after informed written consent according to the Declaration of Helsinki and approved by the Local Ethics Committee (De Videnskabsetiske Komitéer for Region Midtjylland, project numbers 20070008 and 20121329) and the Danish Data Protection Agency.

### Sample handling

Plasma samples were collected in ethylenediaminetetraacetic acid (EDTA) tubes and kept at −80 °C until needed. The PBMCs and SFMCs were isolated by conventional Ficoll-Paque (GE Healthcare) density-gradient centrifugation and cryopreserved at −135 °C until needed.

### IL-19, IL-20, and IL-24 enzyme-linked immunosorbent assays

The plasma concentrations of IL-19, IL-20, and IL-24 were quantified as previously described [25, 33]. Briefly, the three cytokines were measured with commercially available enzyme-linked immunosorbent assay (ELISA) kits (R&D Systems). The ELISA systems used were validated to prevent unspecific binding caused by RF and heterophilic antibodies [33]. The detection limit of the IL-19, IL-20, and IL-24 ELISA systems were 62.5 pg/ml, 62.5 pg/ml, and 31.25 pg/ml, respectively. All plasma samples were diluted 1:3 in blocking buffer; therefore, the cut-offs for the plasma analyses were 187.5 pg/ml, 187.5 pg/ml, and 93.75 pg/ml, respectively.

### Stimulation of PBMCs with ICs

The PBMCs were thawed and cultured in RPMI medium supplemented with 10 % fetal calf serum (FCS), penicillin, streptomycin, and glutamine at a density of 2 × 10^6^ cells/ml. Then two different experimental setups were performed to stimulate the cells with ICs. First, ICs were generated by heat-aggregating human Ig (Behring) for 30 minutes at 65 °C as previously described [34]. Then, 48-well culture plates were coated with either increasing concentrations of the heat-aggregated Ig ICs (haIg-ICs) or with native Ig in phosphate-buffered saline (PBS). For each type of experiment, an untreated (UT) cell culture with the same number of cells in medium without stimulants was used for comparison, and a culture stimulated with lipopolysaccharide (LPS; Sigma-Aldrich) at a concentration of 100 ng/ml was used as a positive control. Cells were cultured for 48 hours at 37 °C in a humidified incubator with 5 % CO_2_ without change of medium. Next, ICs containing cFb were used as previously described [12]. Briefly, ICs were generated by incubating cFb (50 μg/ml) with polyclonal anti-Fb antibody (75 μg/ml) for 45 minutes at 37 °C. Human monocyte-derived macrophages from two HC donors were pretreated for 30 minutes with 1 μg/ml of the TLR4 inhibitor CLI-095 (InvivoGen) and/or 10 μg/ml FcγRIIa-blocking antibody (IV.3; Stem Cell Technologies). Cells were added to 96-well plates coated with the cFb-containing ICs (cFb-ICs) in triplicate and incubated for 24 hours at 37 °C in a humidified incubator with 5 % CO_2_, without change of medium. For comparison, an UT cell culture of the same cells and medium was used. After incubation, supernatants from the triplicates from both donors were pooled. All supernatants were stored at −80 °C until analysis of IL-19, IL-20, and IL-24 content by ELISA.

### IL-20R1 and IL-22R1 flow cytometry

Healthy control PBMCs and RA PBMCs and SFMCs were transferred to FACS tubes (Nunc) in PBS with 0.5 % bovine serum albumin (BSA; Calbiochem) and 0.09 % NaN_3_ together with 100 μg/ml murine gamma globulin (Jackson ImmunoResearch) and 100 μg/ml human Ig (Behring) at room temperature (RT) for 30 minutes to prevent unspecific binding. Receptor expression was analyzed using anti-IL-20R1 PE (173714; R&D Systems), anti-IL-22R1 APC (305405; R&D Systems), live/dead near-IR (Invitrogen), anti-CD14 V500 (MϕP9; BD Biosciences), anti-CD16 FITC (3G8; Bechman Coulter), anti-CD33 PC7 (D3HL60.251; Bechman Coulter), and anti-RANK PerCP (64C1385.1; Novus Biologicals). Fluorescence minus one staining with isotype antibodies served as the negative controls. Cells were incubated with antibodies at RT for 30 minutes. All samples were analyzed within 24 hours using an LSR Fortessa (BD Biosciences) and FlowJo software version 10.1 (Tree Star Inc.). First, cells were gated according to the monocyte population on a plot with forward scatter versus side scatter. Then, dead cells were excluded followed by the selection of singlets. Finally, the CD14-positive cells were selected for further analysis of IL-20R1 and IL-22R1 expression. The IL-20R1 and IL-22R1 positive cells were then studied for co-expression of CD16, CD33, and RANK.

### Generation of OCs from RA SFMCs

Osteoclasts were grown from RA synovial cells as previously described [17]. Briefly, SFMCs were thawed and cultured in Dulbecco’s modified Eagle’s medium (DMEM) supplemented with 10 % FCS, penicillin, streptomycin, and glutamine at a density of 1 × 10^6^ cells/ml at 37 °C in a humidified incubator with 5 % CO_2_. The medium was changed every 3–4 days. Differentiated OCs were used after 19 days of culture for immunofluorescence or the stimulation assay.

### Immunofluorescence of OCs

OCs were grown from RA SFMCs on sterile glass slides in 24-well cell culture plates and stained for confocal microscopy as previously described [35]. Briefly, cells were fixed with 4 % paraformaldehyde for 10 minutes at RT. Non-specific binding was blocked by incubating in PBS with 0.5 % BSA and 5 % goat serum for 30 minutes at RT. Cells were stained with either anti-IL-20R1 IgG1 (173714; R&D Systems) or anti-IL-22R1 IgG1 (305405; R&D Systems) in combination with goat anti-mouse IgG1 Alexa 488 (Invitrogen). Cells were co-stained with anti-TRAP IgG2b in combination with goat anti-mouse IgG2b Alexa 647 (Invitrogen). Isotypes served as negative controls. Glass slides were placed in Prolong Gold Antifade Mountant with DAPI (Life Technologies) and allowed to dry overnight. All micrographs were collected using a Zeiss LSM-710 confocal microscope.

### Stimulation of OCs

Two different experimental setups were carried out to evaluate the effect of the IL-20R cytokines on OCs. First, RA SFMCs were grown with 200 ng/ml IL-19, IL-20, or IL-24 (all R&D Systems). The medium was changed and cells were re-stimulated every 3–4 days to study the effect of the three cytokines on osteoclastogenesis. Second, OCs were generated from RA SFMCs in medium alone for 19 days and then cultured with IL-19, IL-20, and IL-24 (200 ng/ml) at 37 °C in a humidified incubator with 5 % CO_2_ for 48 hours. In all experiments UT cells were used for comparison and cells stimulated with a combination of macrophage-colony stimulating factor (M-CSF; 25 ng/ml) and RANK ligand (RANKL; 50 ng/ml) were used as a positive control. Supernatants were harvested after centrifugation of the culture plates at 1200 rpm for 5 minutes and analyzed for TRAP by enzymatic assay (B-bridge International) and MCP-1 by ELISA (Biolegend).

### Statistics

Statistical analyses were performed using GraphPad Prism 6.0 for Mac (GraphPad Software) and Stata 11.1 for Mac (StataCorp). The plasma concentrations of the IL-20R cytokines, the percentage of receptor-positive monocytes, and the secretion of IL-20R cytokines from PBMCs were not normally distributed even after log transformation. Therefore, these data were analyzed using non-parametric statistics. Groups were compared using the Mann–Whitney U test for non-paired data and the Wilcoxon matched pairs test for paired data. The Friedman test was used to compare three or more groups. Correlations were made using Spearman’s Rho. Multiple regression models were made with the baseline plasma cytokine levels and radiographic progression correcting for age, gender, and disease duration after checking the model assumptions including the distribution of the residuals. The secretion of TRAP and MCP-1 from SFMC-derived OC cultures was calculated as ratios comparing stimulated cells with untreated cells because of donor variation. The ratios were log transformed and analyzed with paired *t*-test. In all tests, the level of significance was a two-sided *P* value of less than 0.05.

## Results

### Plasma concentrations of IL-20 and IL-24 were increased in early RA patients at baseline compared with HCs, and decreased after 6 months of treatment

**Fig. 1:**
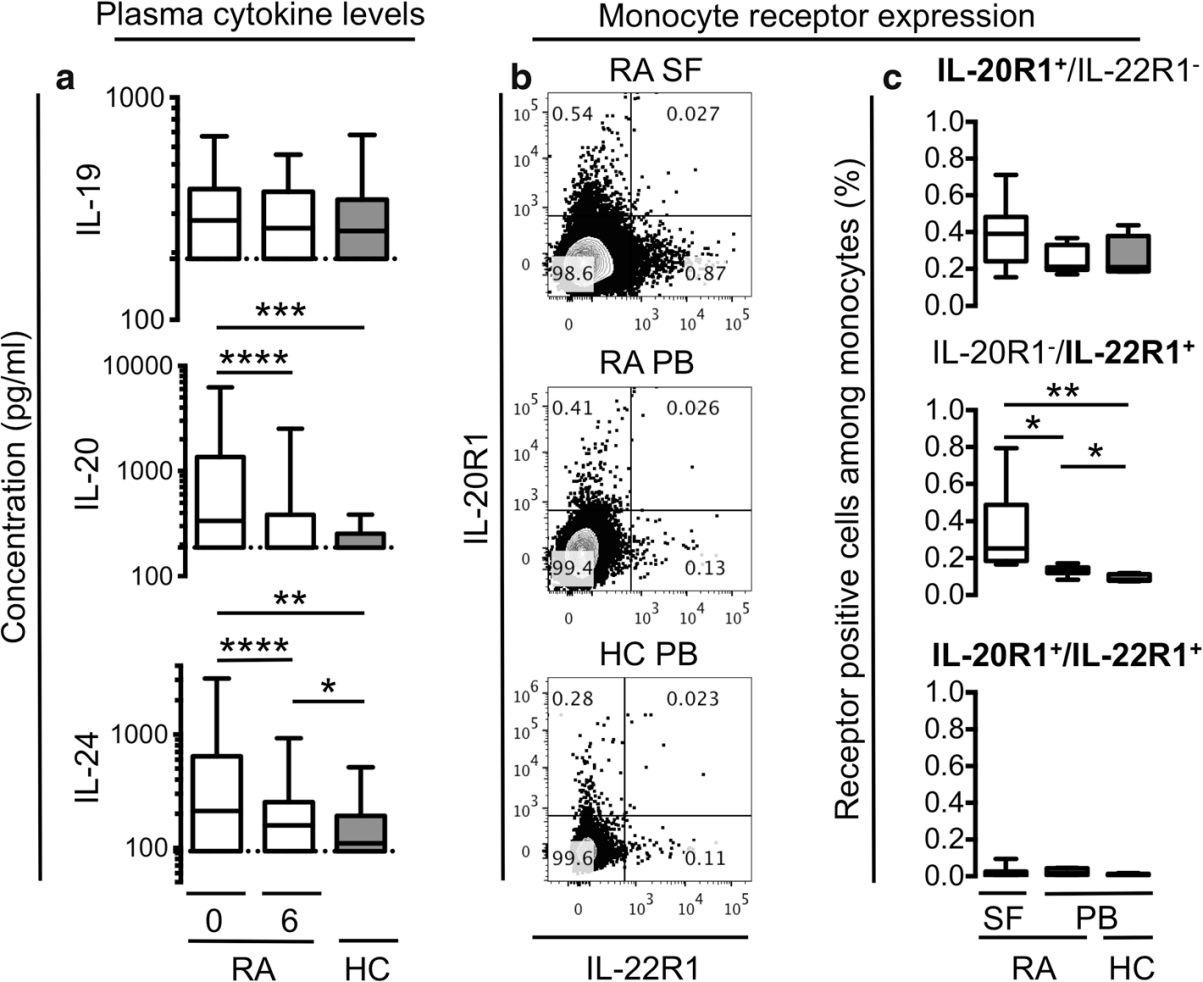
Plasma concentrations of interleukin (*IL*)-19, IL-20, and IL-24 and monocyte expression of IL-20R1 and IL-22R1. **a** The plasma concentrations of IL-20 and IL-24 were increased in early rheumatoid arthritis (*RA*) patients (n = 152) at baseline (*0*) compared with after 6 months of treatment (*6*) and healthy controls (*HCs*) (n = 88). Data were analyzed using the Wilcoxon signed rank test for paired data and the Mann–Whitney U test for unpaired data. **b**, **c** The expression of the IL-22R1 subunit is increased on monocytes from synovial fluid (*SF*) of RA patients compared with the peripheral blood (*PB*) of RA patients and HCs. **b** Representative plots of cell membrane expression of IL-20R1 and IL-22R1 on monocytes from RA SF (n = 7), RA PB (n = 7), and HC PB (n = 5). **c** The percentage of IL-20R1 single positive cells, IL-22R1 single positive cells, and IL-20R1/IL-22R1 double positive cells among monocytes. Monocytes were gated using forward/side scatter and then selecting single cells, live cells, and CD14^+^ cells. Data were analyzed using the Mann–Whitney U test for unpaired data and the Wilcoxon signed rank test for paired data. Boxes indicate the median and interquartile range, and whiskers indicate 10–90 percentiles. *P < 0.05, **P < 0.01, ***P < 0.001, ****P < 0.0001

Patients with early treatment naïve RA and HCs were studied to assess alterations and changes in plasma levels of the IL-20R cytokines before and after a treat-to-target strategy (the OPERA regimen). The plasma concentrations of IL-20 and IL-24 were significantly increased in early RA patients compared with HCs (*P* = 0.0002 and *P* = 0.0016, respectively) and decreased after 6 months of treatment (both *P* < 0.0001) (Fig. 1a). After 6 months of treatment, the plasma concentration of IL-20 decreased to the same level as HCs (*P* = 0.32), while IL-24 remained elevated (*P* = 0.042) (Fig. 1a). The addition of adalimumab to conventional treatment did not further decrease the concentration of IL-20 (*P* = 0.52) or IL-24 (*P* = 0.69). The plasma concentrations of IL-19 did not differ between early RA patients and HCs, and did not change after treatment (Fig. 1a). In summary, IL-20 and IL-24 levels were increased in RA peripheral blood.

### IL-22R1 expression was increased on monocytes from RA SFMCs compared with RA PBMCs and HC PBMCs

Previously, IL-19, IL-20, and IL-24 were found to stimulate monocytes, but the expression of the receptor subunits on these cells has never been studied. Here, the expressions of IL-20R1 and IL-22R1 on RA SFMCs and PBMCs and HC PBMCs were studied by flow cytometry. Monocytes were found among SFMCs and PBMCs using gates on live cells, singlets, and CD14^+^ cells (data available on request). Small subsets of monocytes from RA SFMCs, RA PBMCs, and HC PBMCs expressed IL-20R1 and IL-22R1 (Fig. 1b). The percentage of IL-22R1^+^ cells was increased among RA SF monocytes compared with RA and HC PB monocytes (*P* = 0.016 and *P* = 0.0025, respectively) (Fig. 1c). Furthermore, the percentage of IL-22R1^+^ cells was increased among RA PB monocytes compared with HC PB monocytes (*P* = 0.018) (Fig. 1c). The monocyte expression of IL-20R1 did not differ significantly between RA patients and HCs (Fig. 1c). Our data indicate that the IL-22R1 subunit is increased in RA and found only on a small subset of monocytes.

### Plasma concentrations of IL-20 and IL-24 associated with IgM-RF and anti-CCP positivity in early RA patients

**Fig. 2:**
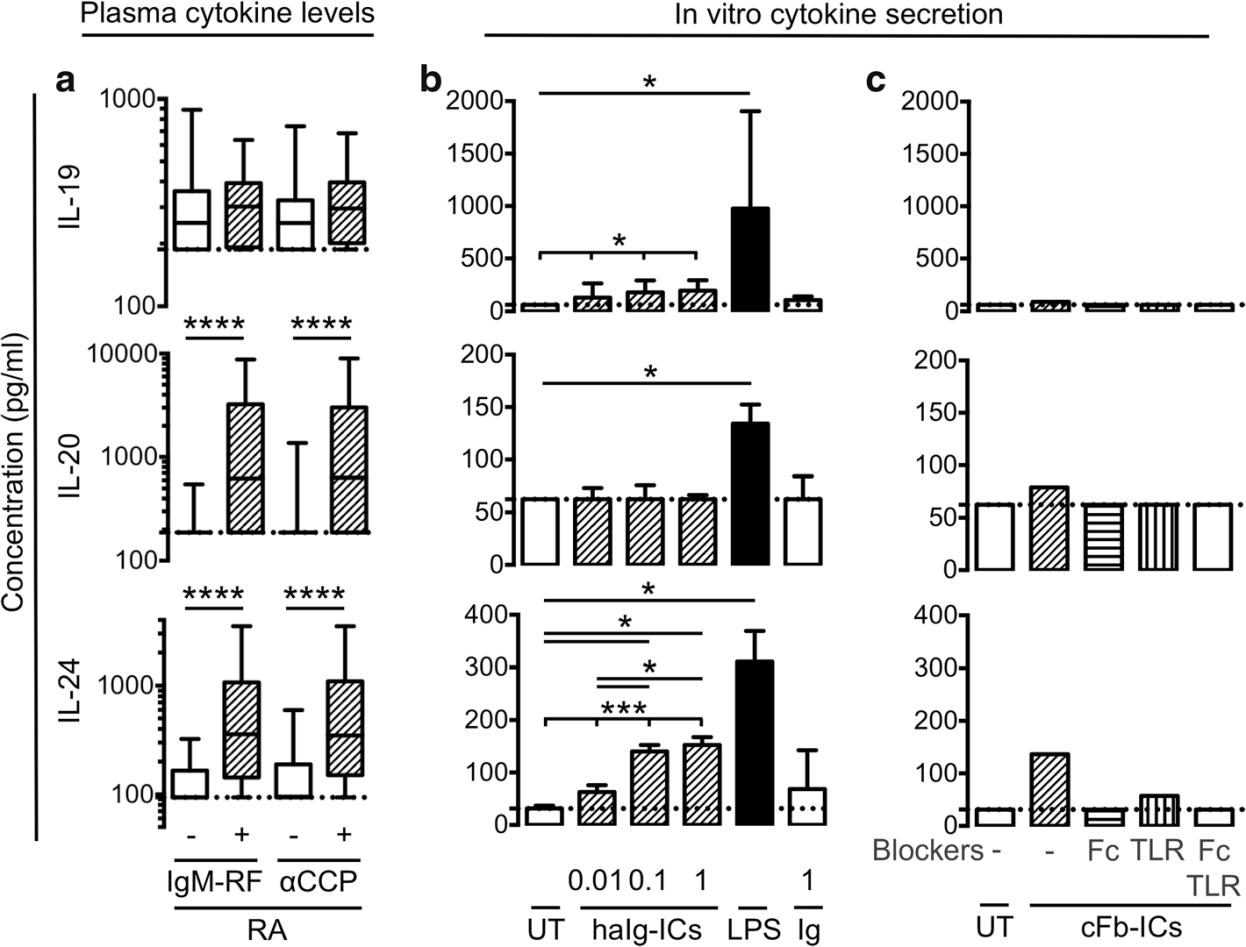
Association of baseline plasma concentrations of interleukin (*IL*)-19, IL-20, and IL-24 with immunoglobulin M-rheumatoid factor (*IgM-RF*) and anti-cyclic citrullinated peptide antibody (*αCCP*) positivity in early rheumatoid arthritis (*RA*) patients and immune complex (*IC*) stimulation of the three cytokines in monocytes/macrophages. **a** The plasma concentrations of IL-20 and IL-24 were increased in IgM-RF positive (n = 108) compared with negative (n = 44) RA patients and in anti-CCP antibody positive (n = 98) compared with negative RA patients (n = 54). Data were analyzed using the Mann–Whitney U test for unpaired data. Boxes indicate the median and interquartile range and whiskers indicate 10–90 percentiles. **b** Secretion of IL-19, IL-20, and IL-24 from HC PBMCs stimulated with heat -aggregated immunoglobulin immune complexes (*haIg-ICs*) at 0.01–1 μg/ml, lipopolysaccharide (*LPS*) at 100 ng/ml, or immunoglobulin (*Ig*) at 1 μg/ml (n = 7). Data were analyzed using the Friedman test for dose–response data and the Wilcoxon signed rank test for paired data. **c** Secretion of IL-19, IL-20, and IL-24 from monocyte-derived macrophages stimulated with citrullinated fibrinogen immune complexes (*cFb-ICs*) with and without the Toll-like receptor 4 inhibitor CLI-095 (*TLR*), Fcγ receptor IIa-blocking antibody (*Fc*), or both inhibitors (n = 2 in triplicates, supernatants pooled). Bars indicate the median and whiskers indicate interquartile range. *P < 0.05, **P < 0.01, ***P < 0.001, ****P < 0.0001

To further elucidate the role of the IL-20R cytokines in RA, associations between plasma concentrations of the three cytokines and the baseline characteristics of age, gender, disease duration, IgM-RF positivity, and anti-cyclic citrullinated peptide (anti-CCP) positivity were analyzed. In early RA patients, the plasma concentrations of IL-20 and IL-24 were significantly increased in IgM-RF positive patients compared with negative patients (both *P* < 0.0001) (Fig. 2a) and in anti-CCP antibody positive patients compared with negative patients (both *P* < 0.0001) (Fig. 2a). The plasma concentrations of IL-19, IL-20, and IL-24 did not associate with age, gender, or disease duration. These findings indicate that the IL-20R cytokines are either involved in pathways leading to the production of antibodies or autoantibodies can trigger the secretion of the IL-20R cytokines.

### ICs stimulated the production of the IL-20R cytokines in HC PBMCs

Both RF and ACPAs can form ICs that can potentially stimulate monocytes/macrophages through FcRs. We tested whether ICs could induce the secretion of IL-19, IL-20, and IL-24 in monocytes/macrophages using two different types of ICs. First, ICs were generated by haIg-ICs. These haIg-ICs stimulated the production of IL-19 and IL-24 (*P* = 0.018 and *P* = 0.0003, respectively) (Fig. 2b). The positive control LPS increased the production of IL-19, IL-20, and IL-24 (*P* = 0.016, *P* = 0.031, and *P* = 0.016, respectively) (Fig. 2b). Then, ICs were generated by combining citrullinated fibrinogen and anti-fibrinogen antibodies (cFb-ICs). Previously, these ICs stimulated macrophages through both TLR4 and FcγRIIa. In line with this, the cFb-ICs increased the secretion of all three cytokines and blockers of TLR4 and the FcγRIIa diminished this induction (Fig. 2c). Taken together, Ig aggregates and ICs containing cFb could to some extent induce IL-19, IL-20, and IL-24 secretion in vitro.

### Plasma concentrations of IL-20 and IL-24 at baseline were associated with radiographic progression after 12 and 24 months

**Fig. 3:**
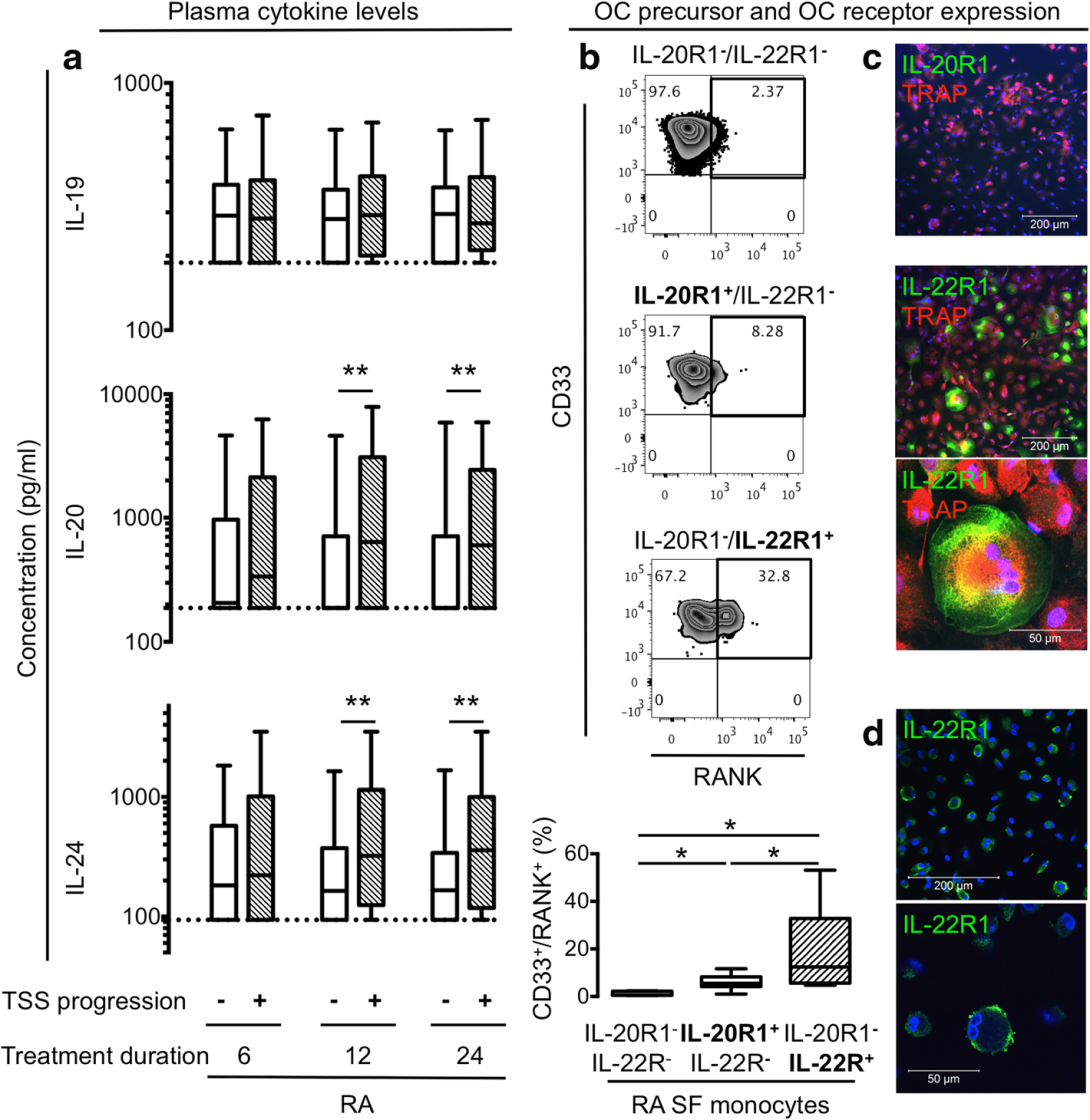
Association of baseline plasma concentrations of interleukin (*IL*)-19, IL-20, and IL-24 and total Sharp score (*TSS*) of radiographic progression in early rheumatoid arthritis (*RA*) patients and IL-20R1 and IL-22R1 expression on osteoclasts (*OCs*) and their precursors. **a** The median baseline values of IL-20 and IL-24 were higher in plasma from patients with TSS progression compared with no progression at 12 and 24 months (see % progressors in Table 1). Data were analyzed using the Mann–Whitney U test. **b** Co-expression of osteoclast precursor markers and IL-20R1 and IL-22R1 on RA synovial fluid (*SF*) monocytes. Representative plots of receptor activator of nuclear factor kappa-B (*RANK*) and CD33 expression on the three monocyte subsets (n = 7). The median percentage of RANK^+^/CD33^+^ cells was increased among IL-20R1^−^/IL-22R1^+^ monocytes compared with IL-20R1^−^/IL-22R1^−^ and IL-20R1^+^/IL-22R1^−^ monocytes. Data were analyzed using the Wilcoxon signed rank test. **c** Representative confocal microscopy images of IL-20R1 and IL-22R1 staining on tartrate-resistant acid phosphatase positive (*TRAP* ^*+*^) cells derived from SFMCs (n = 5). No IL-20R1 expression (20×), and co-expression of TRAP and IL-22R1 in permeabilized cells (20× and 64×, respectively). **d** Surface expression of IL-22R1 on non-permeabilized cells (20× and 64×, respectively). Boxes indicate the median and interquartile range and whiskers indicate 10–90 percentiles. *P < 0.05, **P < 0.01

Correlations between plasma concentrations of IL-19, IL-20, and IL-24 and clinical disease parameters, response rates, and test results obtained through 24 months of follow-up were examined to understand the function of the IL-20R cytokines. We analyzed the patient global, physician global, health assessment questionnaire (HAQ), and disease activity score 28 based on C-reactive protein (DAS28CRP) scores as well as radiographic progression, measured as changes in TSS, erosion score, and joint space narrowing (JSN). The early RA patients with TSS progression after 12 and 24 months had increased baseline plasma concentrations of IL-20 (*P* = 0.0018 and *P* = 0.0047, respectively) and IL-24 (*P* = 0.0077 and *P* = 0.0057, respectively) (Fig. 3a). The association between baseline IL-20 and IL-24 levels and TSS progression did not change after correction for age, gender and disease duration (*P* = 0.07 without correction and *P* = 0.06 with correction for IL-20 and *P* = 0.006 without correction and *P* = 0.005 with correction for IL-24), and was also seen when dividing the TSS into erosion score and joint space narrowing (Table 2). There were no associations between plasma concentrations of IL-19 and radiographic progression (Fig. 3a and Table 2). There were no significant associations between plasma concentrations of the IL-20R cytokines and patient global, physician global, HAQ, and DAS28CRP scores or response rates (Table 2). Also, there were no associations between changes in cytokine levels and disease activity or disease activity improvement. The association between IL-20 and IL-24 and radiographic progression suggests these two cytokines could be involved in bone destruction. In contrast, the IL-20R axis cytokines were not associated with other measures of disease activity.

**Table 2:** Correlations between baseline plasma concentrations of the IL-20R cytokines and clinical parameters, response rates, and radiographic progression

	Baseline cytokine concentration		
	IL-19	IL-20	IL-24
Disease activity scores^a^			
Patient global	0.053 (0.59)	−0.061 (0.46)	0.029 (0.73)
Physician global	−0.044 (0.59)	0.042 (0.61)	0.067 (0.41)
HAQ	−0.013 (0.88)	0.070 (0.39)	0.084 (0.31)
DAS28CRP	−0.015 (0.85)	0.030 (0.72)	0.071 (0.38)
Response rates^b^			
ACR20	−0.093 (0.28)	0.038 (0.66)	0.018 (0.83)
ACR 50	−0.035 (0.69)	0.060 (0.48)	0.038 (0.66)
ACR70	−0.016 (0.85)	0.026 (0.76)	0.055 (0.52)
ACR90	−0.038 (0.66)	−0.021 (0.81)	−0.035 (0.68)
Radiographic progression^c^			
Total Sharp score	0.04 (0.63)	**0.27 (0.001)**	**0.23 (0.005)**
Erosions	−0.014 (0.87)	0.14 (0.094)	**0.17 (0.047)**
Joint space narrowing	0.05 (0.55)	0.16 (0.062)	0.10 (0.23)

Data were analyzed using the Spearman’s correlation. Numbers indicate Spearmans Rho with *P* value in parenthesis. Bold numbers indicate *P* < 0.05. ^a^Disease activity scores at baseline. ^b^Response after 12 months of treatment. ^c^Radiographic progression after 12 months of treatment (12 months – baseline)

*ACR* American College of Rheumatology, *DAS28CRP* disease activity score 28 based on C-reactive protein, *HAQ* health assessment questionnaire, *IL* interleukin

### IL-22R1 was expressed by RANK^+^ OC precursors from RA synovial fluid and in TRAP^+^ multinucleated OCs derived from RA SFMCs

To study the function of IL-20 and IL-24 in bone homeostasis, we first analyzed the expression of IL-20R1 and IL-22R1 on OC precursors and OCs. First, the expression of the surface markers CD16, CD33, and RANK on the IL-20R1^+^ and IL-22R1^+^ monocytes was analyzed by flow cytometry. A large percentage of cells in the IL-22R1^+^ monocyte subset from RA SFMCs co-expressed CD33 and RANK (Fig. 3b). The percentage of CD33^+^ and RANK^+^ monocytes were increased in the IL-22R1^+^ monocytes compared with the IL-20R1^+^ monocytes and the IL-20R1^−^ and IL-22R1^−^ monocytes (both *P* = 0.016) (Fig. 3b). In contrast, there was increased co-expression of CD16 in the IL-20R1^+^ monocyte subset from PBMCs (data available on request). Second, OCs were derived from SFMCs, stained for the expression of IL-20R1 and IL-22R1, and examined by confocal microscopy. Both TRAP^+^ multinucleated OCs and TRAP^+^ single nucleated OC precursors expressed the IL-22R1 subunit, but not IL-20R1 (Fig. 3c and d). These findings point to a role of IL-20 and IL-24 in OC function via the IL-22R1.

### IL-20 and IL-24 increased MCP-1 secretion in OCs derived from RA SFMCs

**Fig. 4:**
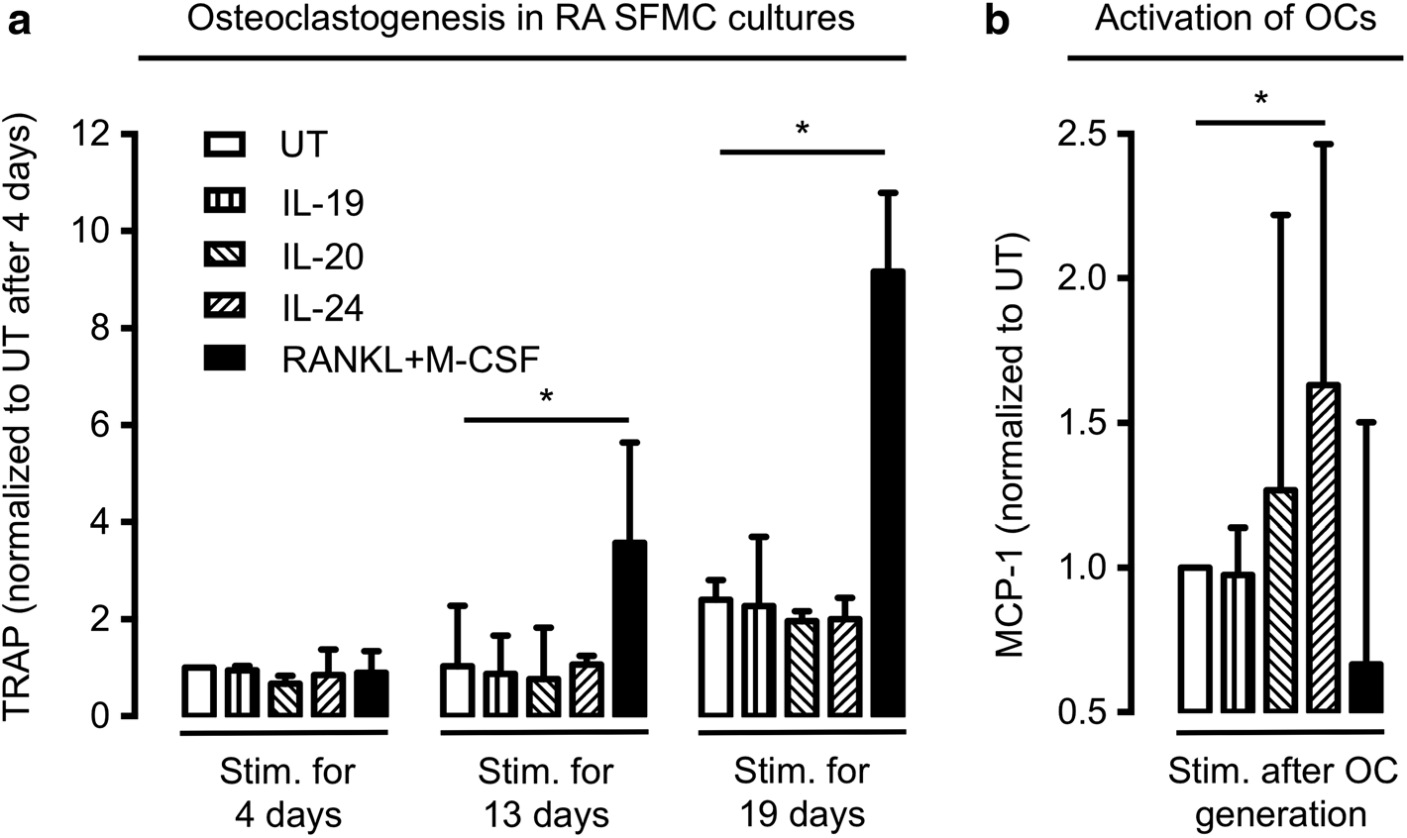
Effect of interleukin (*IL*)-19, IL-20, and IL-24 on osteoclastogenesis and osteoclast (*OC*) MCP-1 secretion. **a** No effect of continuous IL-19, IL-20, and IL-24 stimulation on osteoclastogenesis in cultures of rheumatoid arthritis synovial fluid mononuclear cells (*RA SFMCs*), as measured by tartrate-resistant acid phosphatase (*TRAP*) secretion. **b** IL-20 and IL-24 increased monocyte chemoattractant protein 1 (*MCP-1*) secretion by OCs generated from RA SFMCs prior to stimulation. Positive controls were stimulated with receptor activator of nuclear factor kappa-B ligand (*RANKL*) and macrophage-colony stimulating factor (*M-CSF*) in all experiments. The secretion of TRAP and MCP-1 was calculated as ratios comparing stimulated cells with untreated cells (*UT*). The ratios were then log transformed and analyzed with paired *t*-test. Bars indicate the median and whiskers indicate the interquartile range. *P < 0.05

The functional role of the IL-22R1 on OCs and OC precursors was examined using two different experimental strategies. First, SFMCs were cultured for 19 days with IL-19, IL-20, and IL-24, or a combination of RANKL and M-CSF to study osteoclastogenesis. Second, OCs were generated from SFMCs in medium for 19 days and then stimulated for 48 hours with IL-19, IL-20, and IL-24, or a combination of RANKL and M-CSF. In culture supernatants, IL-20 and IL-24 did not increase osteoclastogenesis, measured by TRAP activity (Fig. 4a). In contrast, IL-20 and IL-24 increased the secretion of MCP-1 in already differentiated OCs (Fig. 4b). Our findings indicate that the membrane-expressed IL-22R1 is part of a functional receptor complex.

## Discussion

In RA, RF and ACPAs are well-known risk factors for the progression of bone erosions [36, 37], but the mechanism for this association is not well understood [38]. Here, we provide evidence for a correlation between the IL-20R axis and progression of structural damage by showing the relationship between IL-20 and IL-24 and RA-associated ICs and OC stimulation via the IL-22R1. Unlike other pro-inflammatory interleukins, the IL-20R cytokines are assumed not to be involved in direct activation of leukocytes [2–4]. Rather, the IL-20R axis is important for barrier functions and tissue homeostasis [4]. This could be interesting in the context of autoimmune disease, because modulation of the IL-20R axis might not result in the increased risk of infection seen with targeting other inflammatory mediators, such as TNFα [5, 6]. The IL-20R axis has already been associated with arthritis and the IL-20R2 subunit was recently identified as a novel RA risk locus [9, 18–23, 25]. However, the cellular sources and targets of the three cytokines and their specific role in disease remain unclear.

Expression of IL-19, IL-20, and IL-24 in both monocytes/macrophages and fibroblast-like synovial cells has been reported in arthritis [18–22, 25]. In our study, two lines of evidence suggest the three cytokines secreted from monocytes/macrophages stimulated by RA-associated ICs could contribute to RA. First, we identified an association between IL-20 and IL-24 plasma levels and IgM-RF and anti-CCP antibody positivity. Second, the production of all three cytokines increased in PBMCs when cultured with ICs and was potentiated by RA-associated ICs capable of co-stimulating TLR4 and FcγRIIa. This increase could be due to both a direct effect of the ICs or an indirect effect of other pro-inflammatory cytokines produced in response to the ICs. Our findings are in line with results from a recent phase IIa trial of anti-IL-20 in RA, where autoantibody-positive patients showed a better response to the anti-IL-20 antibody compared with seronegative patients [28].

Knowledge about the function of the IL-20R axis in RA is limited [4]. Our findings indicate a role of IL-20 and IL-24 in bone homeostasis. First, baseline plasma levels of IL-20 and IL-24 were increased in early RA patients with TSS progression at the follow-up visits at 12 and 24 months. Second, the expression of IL-22R1 was increased on monocytes from the synovial joint and was located to the CD33^+^ and RANK^+^ monocytes. This surface expression pattern is consistent with the IL-22R1-positive cells being primarily OC precursors [15]. This could explain the previous finding of increased MCP-1 secretion by SFMCs in response to IL-20 and IL-24 [20]. Third, IL-22R1 was found on TRAP^+^ multinucleated OCs derived from RA SFMCs. Fourth, IL-20 and IL-24 increased MCP-1 secretion from the differentiated OCs. Taken together, these findings indicate that IL-20 and IL-24 are associated with radiographic progression and that OC precursors and OCs express functional IL-22R1. MCP-1 is a chemokine binding the chemokine receptor CCR2, which is expressed by OC precursors [14, 16]. Thus, we speculate that IL-20 and IL-24 induce MCP-1 production in IL-22R1^+^ OC precursors and OCs without affecting other monocytes. This could attract more OC precursors to sites of ongoing bone degradation. Our findings are in line with the already identified role of IL-20 in osteoporosis [26] and the established association between the IL-22-IL-22R system and bone degradation in RA [39, 40]. However, in this study there was no direct effect of the IL-20R cytokines on osteoclastogenesis. An effect of the IL-20R cytokines on fibroblast-like synovial cells and neutrophil granulocytes cannot be excluded. These cells have been suggested to be one of the drivers of joint space narrowing [41]. In this way, secretion of metalloproteinases from fibroblast-like synovial cells and neutrophil granulocytes could contribute to the association between IL-20 and IL-24 baseline levels and progression in joint space narrowing found in this study.

There were no correlations between the two cytokines and any disease activity score reflecting inflammation at either baseline or follow-up after 6 months of treatment, which suggests that the IL-20R cytokines are not closely linked with inflammation. Also, the general expression of the receptor subunits was rather low among the total monocyte population, suggesting that these cytokines might not contribute to the general activation of monocytes. This is in agreement with a recent comprehensive study of skin infections, where the IL-20R cytokines actually had anti-inflammatory functions [42]. However, the IL-20R cytokines are expressed by both monocytes and fibroblast-like synovial cells in response to pro-inflammatory factors such as IL-1 and TLR4 agonists [4, 25]. Such pro-inflammatory cytokines and molecules decrease with anti-inflammatory treatment. Also, the formation of immune complexes and the expression of fc receptors on monocytes seem to be altered with anti-inflammatory treatment [43]. This could explain the decrease in IL-20 and IL-24 plasma levels seen after anti-inflammatory treatment in this study. In this way, an association between the IL-20R cytokines and inflammation cannot be excluded.

In this study, plasma IL-19 levels were not increased in RA patients compared with HCs and did not associate with radiographic progression. In addition, the percentage of IL-20R1^+^ monocytes was not increased in RA patients compared with HCs; IL-20R1 was not found on OCs; and IL-19 did not activate these cells. Thus, IL-19 might not be involved in bone destruction in RA. We recently identified inverse correlations between plasma IL-19 levels and disease activity in patients with spondyloarthritis suggesting IL-19 and IL-20R1 have anti-inflammatory properties [25]. However, such significant associations were not observed here.

Bone destruction is a key endpoint of arthritis and inhibiting this process is pivotal in managing RA [38]. A recent clinical study identified an association between RF and radiographic progression that was independent of disease activity [44]. This suggests that inflammatory activity is not required for radiographic progression in RA, with other possible mechanisms linking RA pathogenesis and bone destruction. In line with this, it was recently shown that ACPA can directly induce bone loss [45]. Our findings indicate that IL-20 and IL-24 could be another link between the presence of autoantibodies and radiographic progression.

The aim of this study was to determine the role of the IL-20R axis in early RA. In line with this, this study does not provide information to draw conclusions regarding the IL-20R cytokines as biomarkers of disease progression or the IL-20R axis as a potential new therapeutic target. The rather weak association between baseline cytokine levels and radiographic progression indicates that measuring the IL-20R cytokines in early RA might not have much predictive value. However, the findings in this study could help guide future drug development strategies. Anti-IL-20 has been tested in RA and the IL-20R axis is part of the target of inhibitors of downstream signaling molecules such as Janus kinase 1, Tyrosine kinase 2 and signal transducer and activator of transcription 3 [6, 28, 46]. This study suggests that targeting the IL-20R axis could be a treatment of bone destruction in rheumatic disease. In particular, dual inhibition of IL-20 and IL-24 or inhibition of IL-22R1 could be helpful in seropositive RA.

## Conclusions

In conclusion, we report that IL-20 and IL-24 link RA-associated autoantibodies with radiographic progression via IL-22R1. The similarities between IL-20 and IL-24 imply that dual inhibition of the two cytokines and attenuation of IL-22R1 are potential anti-erosive treatment modalities in seropositive RA.

## Abbreviations

ACPA: Anti-citrullinated protein antibody
BSA: Bovine serum albumin
CCP: Cyclic citrullinated peptide
CD: Cluster of differentiation
cFb: Citrullinated fibrinogen
DAS28CRP: Disease activity score 28 based on C-reactive protein
ELISA: Enzyme-linked immunosorbent assay
FCS: Fetal calf serum
haIg-IC: Heat-aggregating immunoglobulin immune complex
HAQ: Health assessment questionnaire
HC: Healthy control
IC: Immune complex
Ig: Immunoglobulin
IL: Interleukin
LPS: Lipopolysaccharide
MCP-1: Monocyte chemoattractant protein 1
M-CSF: Macrophage-colony stimulating factor
OC: Osteoclast
PB: Peripheral blood
PBMC: Peripheral blood mononuclear cell
PBS: Phosphate-buffered saline
R: Receptor
RA: Rheumatoid arthritis
RANK: Receptor activator of nuclear factor kappa-B
RANKL: Receptor activator of nuclear factor kappa-B ligand
RF: Rheumatoid factor
RT: Room temperature
SF: Synovial fluid
SFMC: Synovial fluid mononuclear cell
TLR4: Toll-like receptor 4
TNFα: Tumor necrosis factor alpha
TRAP: Tartrate-resistant acid phosphatase
TSS: Total Sharp score
UT: Untreated
